# Estimation of inbreeding and identification of regions under heavy selection based on runs of homozygosity in a Large White pig population

**DOI:** 10.1186/s40104-020-00447-0

**Published:** 2020-04-28

**Authors:** Liangyu Shi, Ligang Wang, Jiaxin Liu, Tianyu Deng, Hua Yan, Longchao Zhang, Xin Liu, Hongmei Gao, Xinhua Hou, Lixian Wang, Fuping Zhao

**Affiliations:** Key Laboratory of Animal Genetics, Breeding and Reproduction (poultry) of Ministry of Agricuture, Institute of Animal Sciences, Chinese Academy of Agricultural Sciences, Beijing, 100193 China

**Keywords:** Candidate genes, Inbreeding coefficients, Runs of homozygosity, *Sus scrofa*

## Abstract

**Background:**

Runs of homozygosity (ROHs) are homozygous segments of the genome where the two haplotypes inherited from the parents are identical. The current availability of genotypes for a very large number of single nucleotide polymorphisms (SNPs) is leading to more accurate characterization of ROHs in the whole genome. Here, we investigated the occurrence and distribution of ROHs in 3,692 Large White pigs and compared estimates of inbreeding coefficients calculated based on ROHs (*F*_ROH_), homozygosity (*F*_HOM_), genomic relationship matrix (*F*_GRM_) and pedigree (*F*_PED_). Furthermore, we identified genomic regions with high ROH frequencies and annotated their candidate genes.

**Results:**

In total, 176,182 ROHs were identified from 3,569 animals, and all individuals displayed at least one ROH longer than 1 Mb. The ROHs identified were unevenly distributed on the autosomes. The highest and lowest coverages of *Sus scrofa* chromosomes (SSC) by ROH were on SSC14 and SSC13, respectively. The highest pairwise correlation among the different inbreeding coefficient estimates was 0.95 between *F*_ROH_total_ and *F*_HOM_, while the lowest was − 0.083 between *F*_GRM_ and *F*_PED_. The correlations between *F*_PED_ and *F*_ROH_ using four classes of ROH lengths ranged from 0.18 to 0.37 and increased with increasing ROH length, except for ROH > 10 Mb. Twelve ROH islands were located on four chromosomes (SSC1, 4, 6 and 14). These ROH islands harboured genes associated with reproduction, muscular development, fat deposition and adaptation, such as *SIRT1*, *MYPN*, *SETDB1* and *PSMD4*.

**Conclusion:**

*F*_ROH_ can be used to accurately assess individual inbreeding levels compared to other inbreeding coefficient estimators. In the absence of pedigree records, *F*_ROH_ can provide an alternative to inbreeding estimates. Our findings can be used not only to effectively increase the response to selection by appropriately managing the rate of inbreeding and minimizing the negative effects of inbreeding depression but also to help detect genomic regions with an effect on traits under selection.

## Background

The inheritance of identical haplotypes from a common ancestor creates long tracts of homozygous genotypes known as runs of homozygosity (ROHs) [1, 2]. Longer haplotypes are inherited from recent common ancestors, and shorter haplotypes are inherited from distant ancestors [2]. Hence, ROHs can provide accurate predictions of alleles at loci that are identical by descent (IBD) and has been widely applied to accurately estimate levels of autozygosity, which is the homozygous state of IBD alleles in human and livestock populations [2, 3]. There are several factors that increase ROH in a population, such as genetic drift, population bottlenecks, mating of close relatives, and natural and artificial selection [2, 3]. Therefore, we can assess the ROH patterns in one population to understand its demographic history and decipher the genetic architecture of economically important traits.

On the basis of levels of autozygosity, ROHs can be used to estimate the inbreeding coefficient of an individual (*F*), which is defined as the probability that both alleles at any locus within an individual are IBD [4]. Inbreeding leads to harmful effects, such as a reduction in genetic variance and higher frequencies of homozygosity for recessive detrimental mutations, reduction in individual performance (inbreeding depression) and lower population viability [5]. Since inbreeding has been implicated in reduced fitness, there is growing interest in characterizing and monitoring autozygosity to allow accurate estimation of *F*. Traditionally, *F* has been estimated based on pedigree information (*F*_PED_). Hence, the estimation of *F*_PED_ relied strongly on the accuracy and amount of pedigree data available [6]. Currently, genotyping technology is no longer a limiting factor in marker-based studies of inbreeding [7]. The inbreeding estimates can be directly derived from the genomic relationship matrix (*F*_GRM_) using genome-wide single nucleotide polymorphism (SNP) data [8]. Genomic *F* can also measure homozygosity (*F*_HOM_) directly and, thus, can accurately reflect the actual percentage of the genome that is homozygous. Furthermore, genomic *F* allows us to estimate inbreeding and inbreeding depression for specific genomic regions, which is not possible with *F*_PED_. In addition, genomic *F* can be estimated in populations without pedigree information [9].

When computing the more accurate genomic *F* using genotypic data, there is a need to distinguish between alleles that are IBD or identical by state (IBS). This distinction is not easy to make when single markers are analysed, but the use of ROH allows it. Hence, *F*_GRM_ and *F*_HOM_ always overestimate inbreeding levels compared to *F*_PED_ [9], and an alternate approach is to use estimates that are obtained from observed ROHs.

The objective of this study was to identify and characterize ROH patterns in a Large White pig population. We further computed the inbreeding coefficients based on the ROHs identified (*F*_ROH_) and estimated their correlations with those from other methods, including genomic relationship matrix (*F*_GRM_), homozygous coefficients (*F*_HOM_) and pedigree-based coefficients (*F*_PED_). Moreover, ROH islands may have occurred due to selection for functionally important traits.

## Methods

### Ethics statement

All animals were treated following the guidelines established by the Council of China for Animal Welfare. The experimental protocols were approved by the Science Research Department of the Institute of Animal Sciences, Chinese Academy of Agricultural Sciences (CAAS) (Beijing, China).

### Animals and genotyping data

In this study, ear tissue samples were collected from 3,692 Large White pigs from two commercial companies. In these two companies, there were 1,466 and 2,226 pigs, respectively. All samples were genotyped with the GeneSeek GGP Porcine HD array. The SNP chip comprises 50,915 probes that are distributed across 18 autosomes and two sex chromosomes according to the *Sus scrofa 10.2* genome version. To update the SNP positions, we reordered the SNPs according to the newest version of the pig genome, *Sus scrofa 11.1*. Following this, 34,689 autosomal SNPs remained, with a mean distance of 67.052 kb between adjacent SNPs.

### Quality filtering

Quality control was performed using PLINK v1.90 software [10] according to the following criteria: (1) the call rate was higher than 0.9; (2) the minor allele frequency (MAF) was higher than 0.01; and (3) SNPs were filtered to exclude loci assigned to unmapped contigs and to sex chromosomes. After quality control, 3,569 pigs and 33,723 variants were retained.

### Effective population size

The effective population size (*Ne*) was calculated from linkage disequilibrium (LD) [11] using the following equation:

$$ {N}_{T\;(t)}=\frac{1}{\left(4\;f\;\left({c}_t\right)\right)}\;\left(\frac{1}{E\left[{r}_{adj}^2\;|\;{c}_t\right]}-\alpha \right) $$

where *N*_*T*(*t*)_ is the effective population size estimated *t* generations in the past, *c*_*t*_ is the recombination rate defined for a specific physical distance between SNP markers *t* generations in the past, *f*(*c*_*t*_) is the Haldane mapping function of the genetic distance in Morgans between SNPs, ${r}_{adj}^2$ is the linkage disequilibrium estimation after adjusting for sampling bias, and α = {1, 2, 2.2} is a correction for the occurrence of mutations [12].

In this study, *Ne* was computed by implementing *SNeP* software [12]. Considering the relatively small number of SNPs per chromosome [13], we used an α = 2 and estimated the recombination rate between a pair of genetic markers according to Sved and Feldman [14]. The minimum and maximum distances used between SNPs for *Ne* estimation were 0.05 and 5 Mb, respectively. The other parameters were set by default [12].

### Runs of homozygosity detection and classification

ROHs were identified for each individual using PLINK v1.90 software [10], which uses a sliding window approach to scan each individual’s genotype at each marker position to detect homozygous segments [15]. To define a ROH, the following criteria must be fulfilled: (1) a minimum ROH length of 1 Mb; (2) a minimum of 50 consecutive SNPs included in a ROH, which was calculated using the equation proposed by Lencz et al. [16]:

$$ l=\frac{\log_e\frac{\alpha }{n_s\times {n}_i}}{\log_e\left(1-\overline{het}\right)} $$

where α is the percentage of false positive ROHs (set to 0.05 in the present study), *n*_*s*_ is the number of SNPs per individual, *n*_*i*_ is the number of individuals, and $\overline{het}$ is the heterozygosity across all SNPs; (3) a maximum gap between consecutive SNPs of 1 Mb; (4) a minimum density of one SNP in 100 kb; (5) a sliding window of 50 SNPs across the genome that moves one SNP at a time; (6) a maximum of five missing genotypes and one heterozygous genotype in a ROH to avoid underestimation of long ROHs; and (7) a window threshold of 0.01.

In this study, the ROHs identified were further divided into three classes: 1~5, 5~10 and > 10 Mb.

### Inbreeding coefficient estimation

Different estimates of inbreeding coefficients (*F*) were used for all animals:

1. The genealogical inbreeding coefficients (*F*_PED_) were computed for 3,569 pigs using pedigree information recorded between 2011 and 2019. For all individuals who passed quality control, complete pedigree records were available for 3 to 10 generations with an average depth of 6.86. Pedigree information on a total of 7,572 animals between 2011 and 2019 was available. The *F*_*PED*_ was estimated for datasets (*n* = 3,569) according to Wright’s coefficient [17] with the R package *pedigreemm* [18].
2. Genomic inbreeding based on homozygous SNPs was determined using PLINK v1.90 software [10]. The inbreeding coefficient for an individual (*F*_HOM_) was computed as *F*_HOM_ = (*O* − *E*)/(*L* − *E*), where *O* is the number of observed homozygotes, *E* is the number expected by chance, and *L* is the number of genotyped autosomal SNPs.
3. Genomic SNP-by-SNP inbreeding coefficient (*F*_GRM_) estimates were calculated by GCTA software [19]. The *F*_GRM_ was calculated as ${F}_{\mathrm{GRM}}={\sum}_{i=1}^m\left({\left[{\mathrm{x}}_i-E\left({x}_i\right)\right]}^2/\left[2{p}_i\;\left(1-{p}_i\right)\right]-1\right)/m$, where *x*_*i*_ is the number of copies of the reference allele for the *i*^*th*^ SNP, *m* is the number of SNPs, and *p*_*i*_ is the frequency of the reference allele.
4. Genomic inbreeding coefficients were also estimated based on ROHs (*F*_ROH_). The *F*_ROH_ for each animal was calculated as ${F}_{RO H}=\frac{\sum_i{L}_{RO{H}_i}}{L_{auto}}$, where ${L}_{\mathrm{RO}{\mathrm{H}}_i}$ is the length of ROH_*i*_ of individual *i*, and *L*_auto_ is the autosomal genome length covered by the SNPs included in the chip.

The inbreeding coefficients estimated by these four methods were compared using Pearson’s correlation.

### Detection of common runs of homozygosity and gene annotation

To identify the genomic regions that were most commonly associated with ROHs, the percentage of occurrences of SNPs in ROHs was calculated by counting the number of times a SNP was detected in those ROHs across individuals. The genomic regions most commonly associated with ROHs were identified by selecting the top 1% of SNPs observed in ROHs. Adjacent SNPs over this threshold were merged into genomic regions called ROH islands [20, 21].

The gene content of the ROH islands was annotated using the annotation database provided by NCBI (ftp://ftp.ncbi.nlm.nih.gov/genomes/refseq/vertebrate_mammalian/Sus_scrofa/latest_assembly_versions/GCF_000003025.6_Sscrofa11.1). The biological function of each annotated gene within the ROH islands was determined through an extensive accurate literature search.

## Results

### Effective population size

**Fig. 1:**
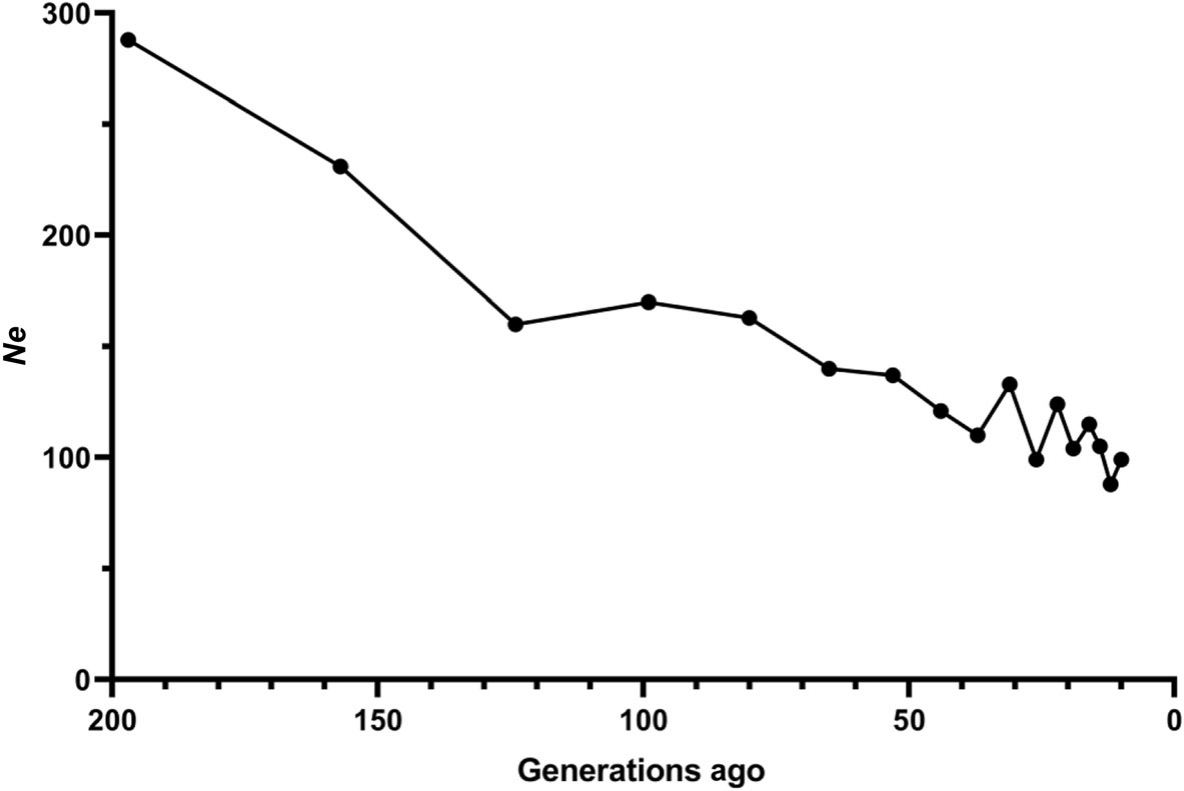
Effective population size (*Ne*) of the Large White pig population

The tendency of effective population sizes (*Ne*) estimated based on linkage disequilibrium (LD) is illustrated in Fig. 1. The historical *Ne* from 197 generations to 10 generations ago of each breed was estimated based on the LD decay. An increasing *Ne* as a function of the number of generations was observed, with a *Ne* of 99 estimated at 10 generations ago and 288 estimated at 197 generations ago.

### Summary of runs of homozygosity

In total, 176,182 ROHs were identified from 3,569 animals. All of the Large White individuals exhibited at least one ROH longer than 1 Mb. Table 1 summarizes the basic statistics of the three classes of ROH. As seen in Table 1, the different classes of ROH length for the genotyped animals revealed that ROHs shorter than 10 Mb predominated. The segments shorter than 10 Mb account for approximately 86.27% of ROHs. Although the number of ROHs 1 ~ 5 Mb was the largest, the proportion of the genome covered by them was relatively small compared with that of ROH segments longer than 10 Mb.

**Table 1:** Descriptive statistics of three classes of runs of homozygosity

Type of ROH	*n*	Number percentage, %	Mean ± SD, Mb	Total length, Mb	Length percentage, %
ROH 1~5 Mb	102,341	58.09	3.24 ± 0.93	331,612.8	29.34
ROH 5~10 Mb	49,645	28.18	6.85 ± 1.35	340,110.2	30.09
ROH > 10 Mb	24,196	13.73	18.95 ± 12.00	458,605.3	40.57

**Fig. 2:**
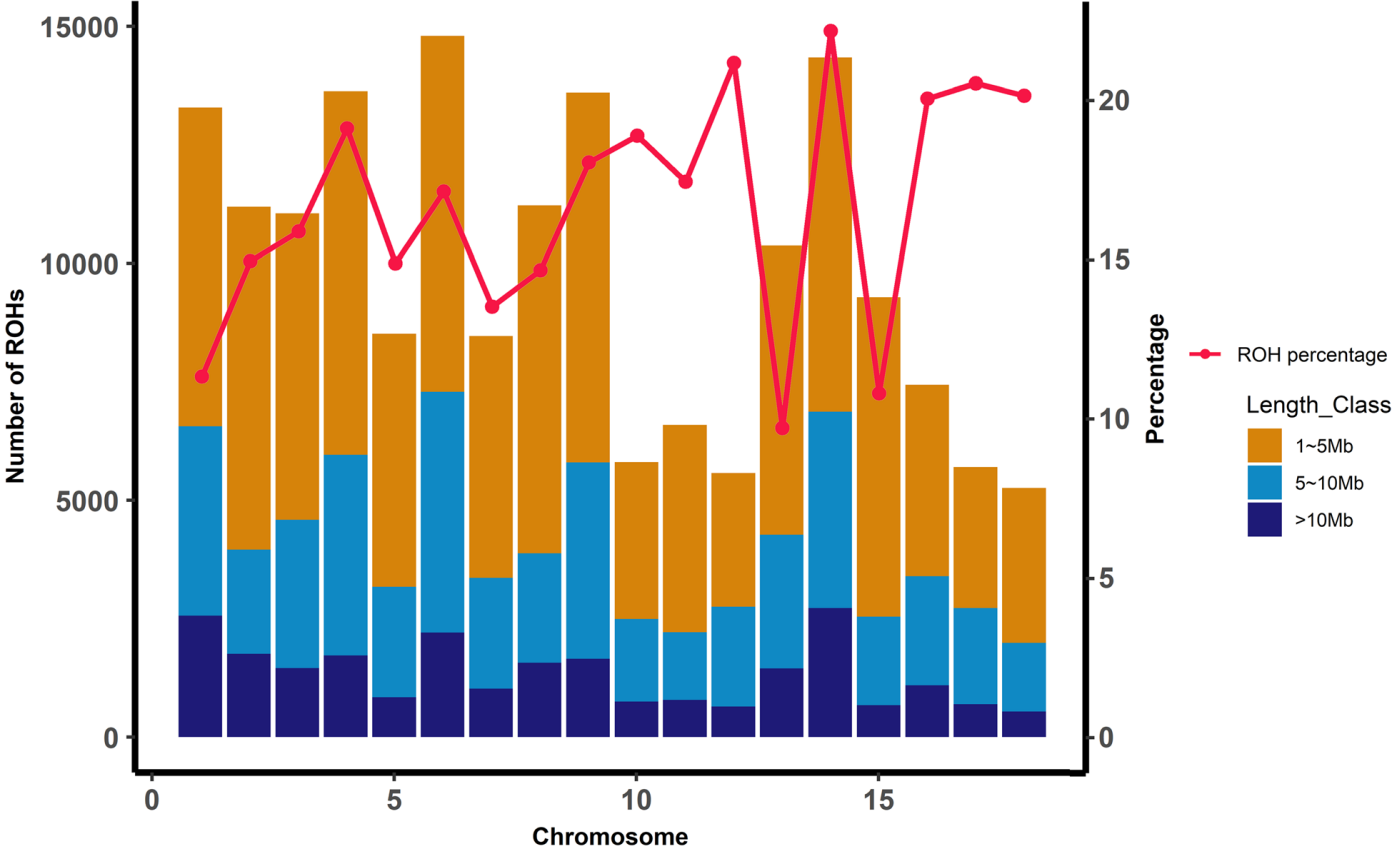
The frequency distribution of the average number of ROHs per chromosome (bars) and average percentage of each chromosome covered by ROHs (lines)

As seen in Fig. 2, the numbers of ROHs on autosomes varied, which indicates that the ROHs identified were unevenly distributed on autosomes. The numbers of ROH per chromosome tended to increase with chromosome length. The smallest number of ROHs per chromosome was found on SSC 12, while the largest number of ROHs was on SSC 6. The highest ROH coverage was observed on SSC14, whereas the lowest was on SSC13 (Fig. 2).

**Fig. 3:**
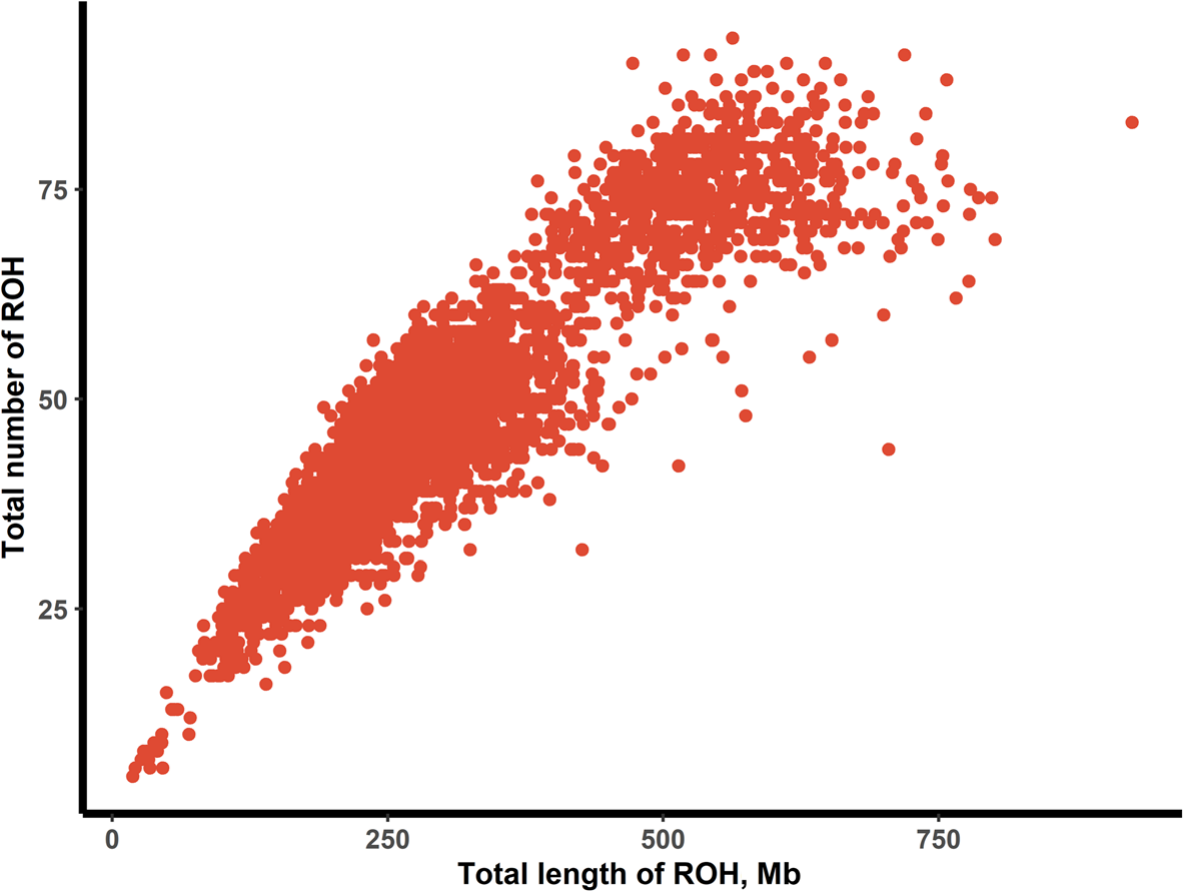
Total number of runs of homozygosity (ROHs) longer than 1 Mb and total length of genome (Mb) covered by ROH segments per individual

The relationship between the number of ROHs and the length of the genome covered by ROHs per individual varied considerably among animals (Fig. 3). As the number of ROHs increased, the cumulative length of the ROHs also increased. In this population, one animal with extremely long ROHs had a length of ~ 900 Mb (925.603 Mb), and one animal with extremely short ROHs had a length of ~ 20 Mb (18.868 Mb).

### Inbreeding coefficient estimates based on pedigree and genomic data

In the present study, seven kinds of inbreeding coefficients were estimated based on the pedigree or genomic data of all individuals. The pedigree-based inbreeding estimates (*F*_PED_) obtained using all pedigree data available ranged from 0 to 0.156 with an average of 0.011. The four kinds of *F*_ROH_ were calculated based on three classes of ROH and the total ROH lengths. The average genomic inbreeding based on the total observed ROHs (*F*_ROH_total_) was 0.140 with a range from 0.008 to 0.409 in this population. The estimated *F*_GRM_ inbreeding coefficients ranged from −0.168 to 1.359 (mean = 0.099), and the estimated *F*_HOM_ inbreeding coefficients ranged from −0.177 to 0.431 (mean = 0.103).

**Fig. 4:**
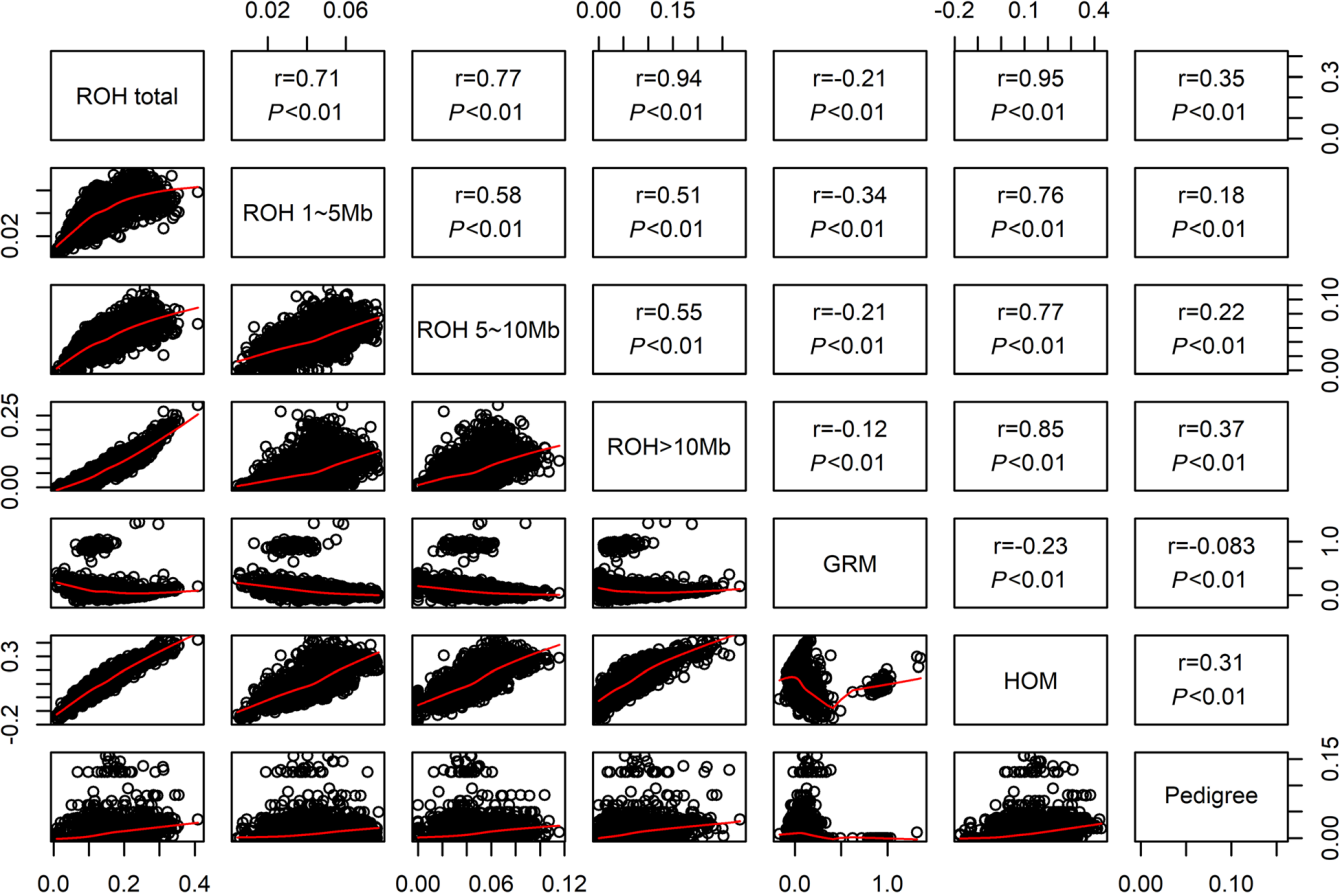
Scatterplots (lower panel) and Pearson’s correlations (upper panel) of the genomic inbreeding coefficients *F*_ROH_ (*F*_ROH_total_, *F*_ROH 1~5 Mb_, *F*_ROH 5~10 Mb_, *F*_ROH > 10 Mb_), *F*_GRM_ and *F*_HOM_, and pedigree-based inbreeding coefficient (*F*_PED_)

Figure 4 depicts the pairwise correlations among seven kinds of inbreeding coefficients. Among all pairwise correlations, the highest correlation was 0.95 between *F*_ROH_total_ and *F*_HOM_, while the lowest correlation was − 0.083 between *F*_GRM_ and *F*_PED_. The correlations between the inbreeding coefficients calculated by different classes of ROH with *F*_PED_ ranged from 0.18 to 0.37, and the highest correlation was found between *F*_ROH > 10 Mb_ and *F*_PED_ (0.37).

### Functional annotation of genes

**Fig. 5:**
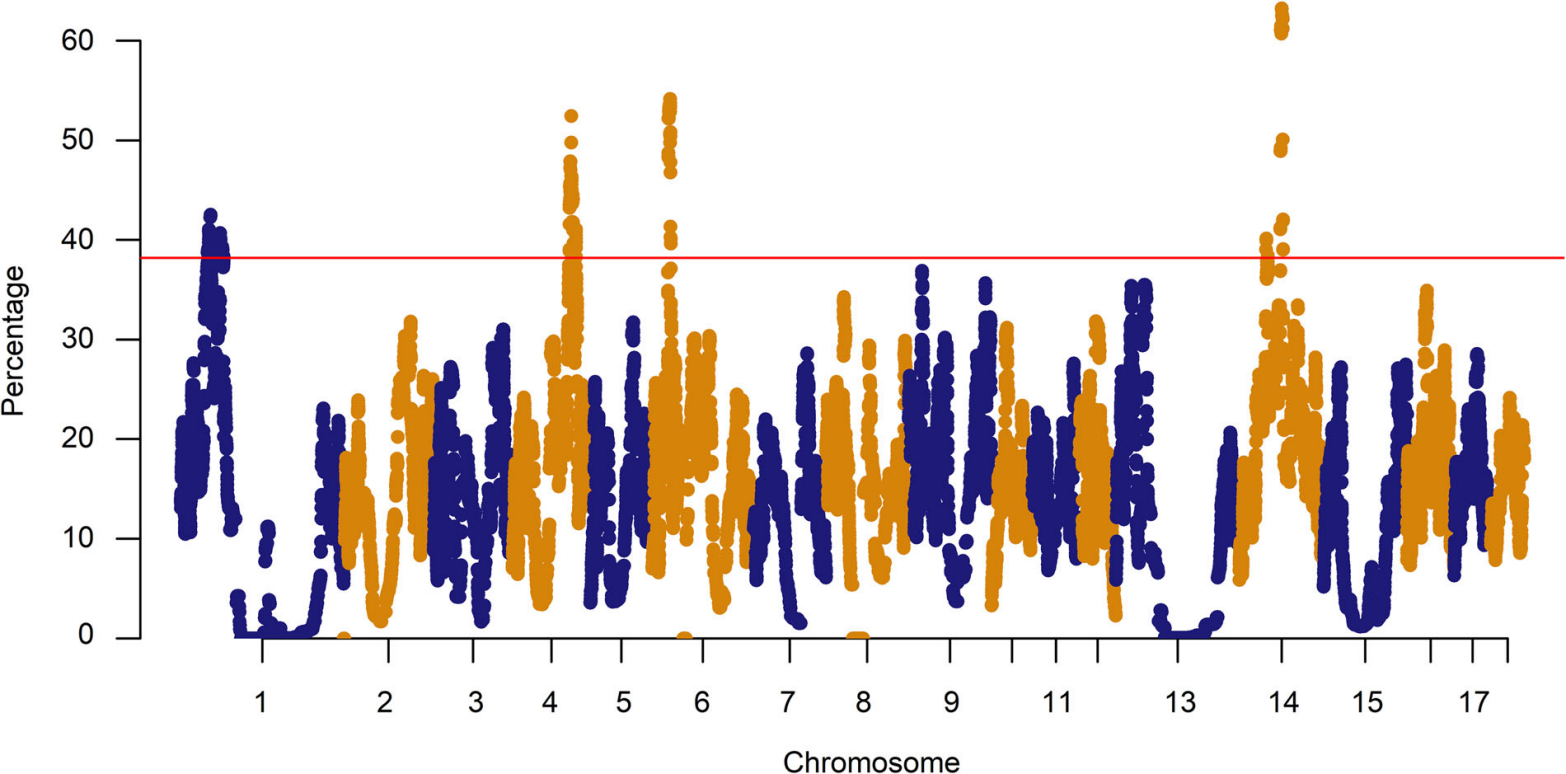
Manhattan plot of occurrences (%) of a SNP in ROHs across individuals

The percentage of SNPs in ROHs was plotted against the positions of the SNPs along the chromosomes in Fig. 5. In this study, the threshold used to define a ROH hotspot in the genome was 38.19%, above which the top 1% of SNPs most commonly observed in ROHs could be selected. The SNP with the highest proportion (63.27% of occurrences) was INRA0044866 on SSC14, which was annotated as within the *SIRT1* gene. *SIRT1* is related to porcine ovarian cell function, suppresses adipogenesis [4, 22], and affects preadipocytes [23]. The region on SSC14 with the second strongest signal harboured one gene: *MYPN* (63.21% of occurrences). The *MYPN* gene has been documented to be associated with meat and carcass traits in Italian Large White pigs [24]. Twelve ROH islands located on four chromosomes (SSC1, 4, 6 and 14) ranged in sizes from 3 SNPs on SSC4 and SSC14 to 107 SNPs on SSC4 (Table 2). These ROH islands harboured important candidate genes, which are shown in Table S1.

**Table 2:** List of genomic regions of extended homozygosity detected in the Large White pig population within each ROH island

Chr.	Start, bp	End, bp	Length, bp	No. SNPs	No. Genes
1	45,159,055	45,580,780	421,725	4	3
1	46,051,365	46,251,692	200,327	4	1
1	47,215,836	49,995,943	2,780,107	50	3
1	66,162,636	67,278,674	1,116,038	18	9
4	96,116,417	102,143,358	6,026,941	107	158
4	102,523,866	102,618,589	94,723	3	1
4	104,299,568	104,397,592	98,024	3	2
4	106,857,958	107,236,227	378,269	12	2
6	26,918,929	31,001,440	4,082,511	71	86
14	44,919,089	45,412,495	493,406	13	3
14	47,355,668	47,467,063	111,395	3	5
14	68,649,520	73,203,453	4,553,933	70	35

*Chr*. chromosome

## Discussion

Traditionally, the inbreeding coefficient was estimated based on pedigree data. In reality, pedigree information might be erroneous, such as having a missing parent or incorrect parent information. Furthermore, Large White pigs in the base population could be hypothesized to be unrelated. Moreover, the *F*_PED_ value is the statistical expectation of the probable IBD genomic proportion [25], and *F*_PED_ does not take into account the stochastic events of recombination during meiosis [26]. Thus, *F*_PED_ could not completely show the actual relatedness among individuals in the population. With the development of high-throughput genotyping technologies and the reduction in genotyping costs, the inbreeding coefficient can be computed based on molecular information [27–29]. Genetic markers can provide a more accurate relationship estimate than pedigree [30]. Since *F*_HOM_ cannot distinguish IBD alleles from IBS alleles, it might overestimate inbreeding levels [31]. In addition, *F*_HOM_ and *F*_GRM_ values can be negative for some individuals. Therefore, using the above three methods to estimate the inbreeding coefficient is not sufficiently accurate. *F*_ROH_ can alleviate the issues mentioned above, and thus, *F*_ROH_ might be a more effective and accurate alternative for quantifying animal relatedness and inbreeding levels in theory [9, 15, 32].

In the present study, the level of inbreeding based on pedigree was expected to be lower than *F*_ROH_ in the Large White pig population. The correlation between the *F*_PED_ and *F*_ROH_ estimates was low (0.18–0.37) (Table 1). These results are consistent with previous studies in other pig populations [33, 34] and Nellore cattle [35]. Pearson’s correlation between *F*_ROH > 10 Mb_ and *F*_PED_ was the highest (0.37) among those between different classes of ROH and *F*_PED_ in the Large White pig population, while the correlation between *F*_PED_ and *F*_ROH_total_ was slightly lower (0.35) (Fig. 4). Compared to the other two classes of ROH, the percentage of ROHs with a length > 10 Mb among ROHs of all lengths was the highest at 40.57%. These results suggested that ROH > 10 Mb was the major contribution to *F*_ROH_total_.

ROHs can reveal the time that inbreeding occurred given the approximate correlation between the length of a ROH and the distance to the common ancestor due to the occurrence of recombination events over time. Fisher [36] reported that the expected length of a DNA segment that is IBD follows an exponential distribution with a mean equal to 1/2 *g* Morgans, where *g* is the number of generations since the common ancestor. Recombination events can interrupt long chromosome segments, so long ROHs (~ 10 Mb) arise as a result of recent inbreeding (up to five generations ago), and short ROHs (~ 1 Mb) are produced by IBD genomic regions from old ancestors [15], which are frequently unaccounted for in the recorded pedigree of an individual.

ROHs can be used to improve the accuracies of genomic breeding values (GEBV). EBVs estimated by the traditional BLUP method are based on pedigrees, which are used to construct a numerator relationship matrix (A matrix). If the A matrix is replaced by a relationship matrix based on genotype data (G matrix), GEBV can be obtained with BLUP. Based on the genotype matrix, it is not possible to identify whether IBS marker alleles are IBD or not. ROHs can identify alleles in the same or different individual(s), which indicates IBD [37]. Luan et al. [38] proposed a novel method to predict GEBV based on ROHs. The results showed that the accuracy of GEBV determination was higher with G_ROH_ than with G_GRM_ by simulation study.

Here, the ROH islands harboured many candidate genes controlling economically important traits of Large White pigs (Table S1). We identified several candidate genes associated with reproduction. *SIRT1* may regulate granulosa cell apoptosis during follicular atresia in porcine ovaries [39] and can reduce ovarian cell viability in rats [40]. *SETDB1* plays an essential role in the maintenance of gonocyte survival in pigs, and knockdown of *SETDB1* can induce gonocyte apoptosis [41]. *PSMD4* effectively inhibits sperm-oocyte binding [42]. *GNRHR2* is involved in regulating reproductive behaviour in pigs [43]. *CES5A*, *GAL3ST1* and *SPAG17* are essential for spermatogenesis and male fertility [44–46]. Some of the candidate genes have been documented as important candidate genes for muscular development and fat deposition. *MYPN* showed an association with traits related to muscularity in Pietrain×(Landrace×Large White) and Duroc × Pietrain, particularly association with ham weight and lean content in Duroc × Pietrain [47]. *SLC12A4* is differentially expressed between the white and red skeletal muscle of Chinese Meishan pigs [48]. *SIRT1* may downregulate pig preadipocyte proliferation and differentiation [23]. *FMO5* plays a role in increasing glucose metabolism and insulin sensitivity in brown adipose tissue [49]. *SIM1* is involved in the regulation of energy homeostasis [50], and *KIF1BP* and *MCHR2* are involved in the regulation of food intake [51, 52], which in turn affects obesity risk [53]. *HORMAD1*, *TBX15* and *WARS2* are also associated with obesity [54, 55]. In addition, *ADGRB3* is related to environmental information processing and environmental adaptation in domestic yak [56].

All the candidate genes residing in ROH islands were further analysed using the DAVID *v*6.8 tool [57] and the *Sus scrofa* annotation file as background to identify significant (*P* < 0.05) GO terms and KEGG pathways. Several GO terms (12 biological process, 5 molecular function and 2 cellular component) were significant, and two were significant for KEGG (Additional file: Table S2). The GO term spermatogenesis (0007283) was of particular note, where there were 6 genes. These results reflected that most quantitative phenotypic traits are likely to be influenced by multiple genes. The enrichment results provide novel insights into the genetic architecture of traits under selection. However, the information provided by GO analysis is limited.

## Conclusions

In this study, we investigated the occurrence and distribution of ROHs on the autosomes of Large White pigs. The number of ROHs shorter than 10 Mb was the highest, while the genome sequence length covered by ROHs was the longest for ROHs longer than 10 Mb. Among the correlations between the genomic inbreeding coefficients calculated by different methods and the correlation coefficient based on pedigree calculation, the correlation between *F*_PED_ and *F*_ROH > 10 Mb_ was the highest. *F*_ROH_ might be an effective and accurate alternative for assessing animal relatedness and inbreeding levels. ROH islands harboured many candidate genes controlling reproductive, muscular development, fat deposition and adaptation. Our findings contribute to an understanding of inbreeding effects when assessing ROHs at the genome level and how selection can shape the distribution of ROH islands in the swine genome.

## Supplementary information

**Additional file 1: Table S1**. Gene content inside run-of-homozygosity islands.

**Additional file 2: Table S2** GO terms and KEGG pathways enriched (*P* < 0.05) based on run-of-homozygosity islands.

## Data Availability

The data and computing programs used in this manuscript are available from the corresponding authors on request.

## Abbreviations

*F*: Inbreeding coefficient
GEBV: Genomic breeding values
GRM: Genomic relationship matrix
HOM: Homozygosity
IBD: Identical by descent
IBS: Identical by state
LD: Linkage disequilibrium
*Ne*: Effective population sizes
PED: Pedigree
ROH: Runs of homozygosity
SNPs: Single nucleotide polymorphisms
SSC: *Sus scrofa* chromosomes
