# Supplementary material for: Estimation of inbreeding and identification of regions under heavy selection based on runs of homozygosity in a Large White pig population
Source: J Anim Sci Biotechnol. 2020 Apr 28;11:46. doi: 10.1186/s40104-020-00447-0 (PMC7187514; doi:10.1186/s40104-020-00447-0)
Supplement: Supplementary file 1 — Additional file 1: Table S1. Gene content inside run-of-homozygosity islands. [file 40104_2020_447_MOESM1_ESM.docx]

Table S1 Genes detected in Large White pig population within each ROH island

| Chr | Start, bp | End, bp | SNPs | Genes |
| --- | --- | --- | --- | --- |
| 1 | 45,159,055 | 45,580,780 | 4 | *PTP4A1*, *PHF3*, *EYS* |
| 1 | 46,051,365 | 46,251,692 | 4 | *EYS* |
| 1 | 47,215,836 | 49,995,943 | 50 | *LOC110259411*, *ADGRB3**, *LMBRD1* |
| 1 | 66,162,636 | 67,278,674 | 18 | *FAXC*, *COQ3*, *PNISR*, *USP45*, *TSTD3*, *CCNC*, *PRDM13*, *MCHR2**, *SIM1** |
| 4 | 96,116,417 | 102,143,358 | 107 | *LOC110260465, S100A7, LOC102161828, S100A8, S100A12, S100A9, PPGRP-S, PGLYRP3, LOR, PRR9, LELP1, LOC110260467, LOC102164580, LOC106510154, LOC100737840, LOC110255231, LOC100157968, SPRP, LOC106510150, IVL, SMCP, KPRP, C4H1orf68, LOC106510156, LOC106510158, LOC106510159, LOC100152299, LOC100153482, CRNN, HRNR, LOC110260322, LOC100513083, RPTN, TCHH, TCHHL1, S100A11, S100A10, THEM5, C2CD4D, RORC, LINGO4, TDRKH, OAZ3, MRPL9, RIIAD1, CELF3, SNX27, TUFT1, CGN, POGZ, PSMB4, SELENBP1, RFX5, PI4KB, ZNF687, PSMD4*, PIP5K1A, VPS72, TMOD4, SCNM1, LYSMD1, TNFAIP8L2, SEMA6C, GABPB2, MLLT11, CDC42SE1, C4H1orf56, BNIPL, PRUNE1, MINDY1, ANXA9, CERS2, SETDB1*, ARNT, CTSK, CTSS, HORMAD1*, GOLPH3L, ENSA, MCL1, ADAMTSL4, ECM1, TARS2, RPRD2, PRPF3, MRPS21, CIART, C4H1orf54, APH1A, CA14, ANP32E, PLEKHO1, VPS45, OTUD7B, MTMR11, SF3B4, SV2A, BOLA1, LOC100154181, LOC106510170, LOC100738859, LOC102161782, LOC100624086, LOC102162202, LOC100738744, LOC102159655, LOC100156741, LOC100621389, LOC110255273, LOC100155404, FCGR1A, LOC100156977, HFE2, TXNIP, POLR3GL, ANKRD34A, LIX1L, RBM8A, GNRHR2*, PEX11B, ITGA10, ANKRD35, PIAS3, NUDT17, POLR3C, RNF115, CD160, PDZK1, LOC100154179, GJA8, GJA5, ACP6, BCL9, LOC110260330, LOC110260331, CHD1L, FMO5*, PRKAB2, LOC100519022, LOC100517759, LOC100624559, LOC100157002, NOTCH2, LOC106505207, LOC102164472, LOC102159389, LOC102159652, ADAM30, REG4, LOC110260333, LOC100156167, HMGCS2, PHGDH, ZNF697, HSD3B1, HAO2, WARS2*, TBX15** |
| 4 | 102,523,866 | 102,618,589 | 3 | *SPAG17** |
| 4 | 104,299,568 | 104,397,592 | 3 | *ATP1A1,* *LOC106508382* |
| 4 | 106,857,958 | 107,236,227 | 12 | *PHTF1*, *MAGI3* |
| 6 | 26,918,929 | 31,001,440 | 71 | *CDH5, BEAN1, LOC110260929, TK2, LOC110260934, LOC110260932, CMTM2, CMTM3, CMTM4, DYNC1LI2, TERB1, NAE1, CA7, PDP2, CDH16, RRAD, FAM96B, CES3, CBFB, C6H16orf70, B3GNT9, TRADD, FBXL8, LOC100523672, KIAA0895L, EXOC3L1, E2F4, ELMO3, MIR328, LRRC29, TMEM208, FHOD1, SLC9A5, PLEKHG4, KCTD19, LRRC36, TPPP3, ZDHHC1, HSD11B2, ATP6V0D1, AGRP, RIPOR1, LOC102161584, CTCF, CARMIL2, ACD, PARD6A, ENKD1, C6H16orf86, GFOD2, RANBP10, TSNAXIP1, CENPT, THAP11, NUTF2, EDC4, NRN1L, PSKH1, CTRL, PSMB10, LCAT, SLC12A4*, LOC100621677, LOC100621778, LOC106507566, DDX28, DUS2, NFATC3, ESRP2, PLA2G15, SLC7A6, SLC7A6OS, PRMT7, SMPD3, OGFOD1, NUDT21, AMFR, GNAO1, CES5A*, CES1, SLC6A2, LPCAT2, CAPNS2, MMP2, IRX6, IRX5* |
| 14 | 44,919,089 | 45,412,495 | 13 | *MN1,* *PITPNB,* *TTC28* |
| 14 | 47,355,668 | 47,467,063 | 3 | *RNF215,* *SEC14L2*, *MTFP1*, *LOC100156470*, *GAL3ST1** |
| 14 | 68,649,520 | 73,203,453 | 70 | *CTNNA3, LRRTM3, DNAJC12, SIRT1*, HERC4, MYPN*, ATOH7, PBLD, HNRNPH3, RUFY2, DNA2, SLC25A16, TET1, CCAR1, STOX1, DDX50, DDX21, KIF1BP*, SRGN, VPS26A, SUPV3L1, HKDC1, HK1, TACR2, TSPAN15, C14H10orf35, NEUROG3, COL13A1, LOC100738475, AIFM2, LOC102161997, TYSND1, SAR1A, PPA1, NPFFR1* |

*commented in the Discussion
