# Supplementary material for: Estimation of inbreeding and identification of regions under heavy selection based on runs of homozygosity in a Large White pig population
Source: J Anim Sci Biotechnol. 2020 Apr 28;11:46. doi: 10.1186/s40104-020-00447-0 (PMC7187514; doi:10.1186/s40104-020-00447-0)
Supplement: Supplementary file 2 — Additional file 2: Table S2 GO terms and KEGG pathways enriched (P < 0.05) based on run-of-homozygosity islands. [file 40104_2020_447_MOESM2_ESM.docx]

Table S2 GO terms and KEGG pathways analysis enriched (*p* < 0.05) based on runs of homozygosity island

| Terms | Genes Count | Gene | P-value |
| --- | --- | --- | --- |
| GO Biological Process |  |  |  |
| GO:0030216~keratinocyte differentiation | 4 | *TXNIP, LOC100157968, SPRP, IVL* | 0.006 |
| GO:0001678~cellular glucose homeostasis | 3 | *HKDC1, HK1, SIRT1* | 0.008 |
| GO:0030574~collagen catabolic process | 3 | *CTSK, CTSS, MMP2* | 0.008 |
| GO:0097193~intrinsic apoptotic signaling pathway | 3 | *LOC100523672, MLLT11, SIRT1* | 0.010 |
| GO:0051603~proteolysis involved in cellular protein catabolic process | 4 | *PSMB10, PSMB4, CTSK, CTSS* | 0.015 |
| GO:0016485~protein processing | 4 | *APH1A, TYSND1, CTSS, SRGN* | 0.024 |
| GO:0007283~spermatogenesis | 6 | *OAZ3, TDRKH, CELF3, HORMAD1, SIRT1, GAL3ST1* | 0.025 |
| GO:0002793~positive regulation of peptide secretion | 2 | *S100A8, S100A9* | 0.028 |
| GO:0070488~neutrophil aggregation | 2 | *S100A8, S100A9* | 0.028 |
| GO:0006334~nucleosome assembly | 4 | *LOC102162202, LOC100738744, LOC100738859, LOC100156741* | 0.038 |
| GO:0001957~intramembranous ossification | 2 | *MMP2, MN1* | 0.042 |
| GO:0008380~RNA splicing | 3 | *RBM8A, CELF3, SCNM1* | 0.044 |
| GO Cellular Component |  |  |  |
| GO:0005737~cytoplasm | 42 | *PSMB10, TNFAIP8L2, TSNAXIP1, FAM96B, REG4, TUFT1, WARS2, ENSA, ARNT, PBLD, LOC100157968, PSMB4, OAZ3, CHD1L, ACD, LRRTM3, RBM8A, LIX1L, OTUD7B, PHTF1, IVL, THAP11, PRUNE1, TXNIP, SETDB1, CAPNS2, SPRP, POGZ, S100A11, C4H1ORF56, PRPF3, PI4KB, SIRT1, PPA1, S100A12, OGFOD1, COQ3, PIAS3, MLLT11, CELF3, HORMAD1, USP45* | 0.007 |
| GO:0001533~cornified envelope | 3 | *LOC100157968, SPRP, IVL* | 0.009 |
| GO:0000786~nucleosome | 4 | *LOC106510170, LOC100156741, LOC100621389, LOC100154181* | 0.018 |
| GO:0000788~nuclear nucleosome | 3 | *LOC102162202, LOC100738744, LOC100738859* | 0.021 |
| GO:0070062~extracellular exosome | 30 | *TPPP3, LOC100156470, S100A8, S100A9, PITPNB, KPRP, SELENBP1, CRNN, PBLD, PSMB4, ANXA9, LCAT, ATP6V0D1, SAR1A, AGRP, LOC100621389, LOC100154181, ZDHHC1, CES3, S100A10, ECM1, PPA1, PEX11B, MTMR11, CDH16, HAO2, NUTF2, LOC100156741, VPS26A, PDZK1* | 0.042 |
| GO Molecular Function |  |  |  |
| GO:0003677~DNA binding | 18 | *SETDB1, LOC106510170, POGZ, AIFM2, LOC100738744, POLR3C, LOC100738859, TET1, ARNT, LOC102162202, CERS2, OTUD7B, CENPT, SUPV3L1, THAP11, LOC100621389, SIM1, LOC100154181* | 2.55×10^-4^ |
| GO:0005509~calcium ion binding | 15 | *CAPNS2, S100A8, S100A7, S100A9, S100A11, S100A10, CRNN, LPCAT2, CDH5, S100A12, NOTCH2, ANXA9, CDH16, RPTN, LOC100523672* | 0.006 |
| KEGG Pathway |  |  |  |
| ssc05322:Systemic lupus erythematosus | 11 | *LOC102159655, LOC102162202, LOC102161782, FCGR1A, LOC100738744, LOC100624086, LOC100738859, LOC100155404, LOC100156741, LOC100621389, LOC100154181* | 2.16×10^-7^ |
| ssc05034:Alcoholism | 11 | *LOC102159655, LOC102162202, GNAO1, LOC102161782, LOC100738744, LOC100624086, LOC100738859, LOC100155404, LOC100156741, LOC100621389, LOC100154181* | 6.46×10^-6^ |
